# Differential associations between body composition indices and neurodevelopment during early life in term-born infants: findings from the Pakistan cohort: Multi-Center Body Composition Reference Study

**DOI:** 10.1038/s41430-023-01296-6

**Published:** 2023-07-12

**Authors:** Shabina Ariff, Almas Aamir, Aneurin Young, Laila Sikanderali, Arjumand Rizvi, Fariha Shaheen, Gul Nawaz Khan, Sajid Soofi, Michelle Fernandes

**Affiliations:** 1 Department of Paediatrics and Child Health, Aga Khan University, Karachi, Pakistan; 2 The Neonatal Intensive Care Unit, Princess Anne Hospital, University Hospital Southampton NHS Foundation Trust, Southampton, UK; 3 MRC Lifecourse Epidemiology Centre and Human Development and Health Academic Unit, Faculty of Medicine, University of Southampton, Southampton, UK; 4 Synergy Groups Medical, Houston, TX USA; 5 Center of Excellence in Women and Children, Aga Khan University, Karachi, Pakistan; 6 Institute of Global Health and Development, Aga Khan University, Karachi, Pakistan; 7 Nuffield Department of Women’s & Reproductive Health, John Radcliffe Hospital, University of Oxford, Oxford, UK

**Keywords:** Nutrition, Paediatrics

## Abstract

**Objective:**

We examined associations between fat free mass (FFM) and fat mass (FM) accretion during the first 1000 days of life and neurodevelopment in term-born, low-risk infants from Karachi, Pakistan.

**Design:**

Prospective, observational study nested within the larger Multi-Center Body Composition Reference Study. FFM, FM, and fat% were estimated using measured deuterium dilution method. Neurodevelopmental outcomes were assessed at 24 months on the INTER-NDA (INTERGROWTH-21st Project Neurodevelopment Assessment) (*n* = 132).

**Results:**

Children with gross motor delays had significantly lower FFM at 18 months (8.01 ± 0.97 kg vs. 7.55 ± 0.20 kg). Children with positive and negative behavior problems had significantly higher fat% at 24 months (20.62 ± 4.30% vs. 18.23 ± 5.46%) and 20.89 ± 4.24% vs. 18.54 ± 5.38%). No associations remained significant after adjusting for covariates. Trajectory modeling showed that between 12 and 18 months, negative behavior scores changed by 13.8 points for every standard deviation change in fat accretion.

**Conclusions:**

Our findings highlight the importance of balancing neurodevelopment and metabolic risk when designing nutritional interventions for young children.

## Introduction

There is robust evidence that the first 1000 days of life are a critical window of both vulnerability and opportunity in the context of growth and neurodevelopment, with pervasive effects on later risk of ill health [1, 2]. Strong associations between growth and neurodevelopment during early childhood have been demonstrated across populations [3, 4], so much so that, in some comparisons, childhood stunting has been employed as a proxy marker for neurodevelopmental risk [3]. This approach is questionable because it is well-established that infants of similar weight, length or even weight-for-length can vary substantially in body composition indices (BCIs) [5]. Additionally, although frequently utilized markers of improved lean and fat growth during early life, namely length gains and increased body mass index (BMI) gain, are associated with improved cognition [6] and later obesity [7] respectively, length and BMI may not accurately reflect lean and fat compartments during infancy and childhood [8].

These observations emphasize the importance of more detailed characterization of BCIs at an early age [9]. However, evidence about how BCI variations impact neurodevelopmental profiles in healthy infants, and whether particular BCI phenotypes are associated with an increased risk of developmental delay during early childhood, is limited. Most evidence linking BCIs with neurocognition in children has emanated from studies of (1) preterm infants [8, 10, 11], (2) children with specific congenital pathologies such as gastrointestinal anomalies and hypothyroidism [12, 13] and (3) populations from high-income countries (HICs) [8, 14, 15]. It is not clear as to how these findings may be generalized to populations of healthy infants from low- and middle-income countries (LMICs).

To address this gap, we studied the associations between fat free mass (FFM) and fat mass (FM) accretion during the first 1000 days of life and neurodevelopment at 24 months in term-born infants, with low-risk indicators for perinatal and postnatal morbidity, from Karachi, Pakistan. Among South Asian countries, Pakistan is ranked as having the highest prevalence of childhood stunting (40.2%), wasting (17.7%) and underweight (28.9%) [16]. The aims of our study were to (1) examine associations between FFM and FM accretion and neurodevelopmental outcomes and (2) compare associations between length, FFM accretion and fat % and developmental delay during early childhood.

## Methods

### Study design and setting

This prospective, observational study was conducted at the Aga Khan University Hospital in Karachi, Pakistan between October 2014 and November 2017. The study was nested within the Multi-Center Body Composition Reference (MBCRS) parent study, whose primary aim was to produce BCI reference data from healthy infants at study centers in Australia, Brazil, Pakistan, India, Sri Lanka and South Africa [17].

Repeated anthropometric and BCI measurements were performed on term-born infants with low-risk indicators for perinatal and postnatal morbidity at 3, 6, 9, 12, 18, and 24 months of age (Table S1). Neurodevelopmental outcomes were measured at 24 months on the INTERGROWTH-21st Project Neurodevelopment Assessment (INTER-NDA) [18].

The anthropometric measurements obtained were weight, length, head circumference (HC), mid-upper arm circumference (MUAC), and triceps (TSF) and subscapular skin fold (SKF) thickness. BCI estimations were represented by FM(kg), FFM(kg) and fat%. The neurodevelopmental outcomes studied were cognitive, language, fine motor, gross motor, positive behavior and negative behavior INTER-NDA scores and the proportions of delays.

### Sample size

The sample size (*n* = 150) was determined based on the primary MBCRS outcomes. This enabled our study to detect FM and FFM for boys and girls <1 SD away from a US-based reference study [19] with a power of 90% [16].

### Participants and eligibility

Mother-infant dyads were enrolled at birth. Mothers aged over 18 years with singleton pregnancies, who had given birth between 37 and 41 gestational weeks and who intended to exclusively breastfeed their infants for the first 6 months of life were included. Those who (1) had not attained at least a secondary level education, (2) were smokers or (3) were known to have a significant morbidity, were excluded.

### Measures

#### BCI measures: the deuterium dilution method (DDM)

DDM is based on the principal of deuterium enrichment in the FM and FFM body compartments [20]. Deuterium is a stable (non-radioactive) isotope of hydrogen. In our study, deuterium was administered orally to infants. Total body water (TBW) and the enrichment of deuterium were quantified in infant saliva through isotope ratio mass spectrometry [21]. FM, FFM and fat% were estimated from TBW [20].

#### Anthropometry

Anthropometric measurements were undertaken by two independent assessors according to the WHO Multi-center Growth Reference Study (MGRS) protocol [22]. All measurements were converted to age and sex adjusted *Z* scores based on the WHO MGRS standards [22].

#### Neurodevelopment: the INTERGROWTH-21st Project Neurodevelopment Assessment (INTER-NDA)

Cognition, language, fine and gross motor, and positive and negative behavior outcomes were measured by assessors trained and standardized in the INTER-NDA (www.inter-nda.com) according to the INTERGROWTH-21st Project’s standardization protocols [18]. The INTER-NDA is a standardized, psychometrically valid, international, rapid developmental assessment for children aged 22 to 30 months [18, 23]. It can be administered reliably by non-specialist assessors in low resource settings with high levels of inter-rater (*k* = 0.70; 95% CI 0.47–0.88) and test–retest reliability (*k* = 0.79; 95% CI 0.48–0.96) [18]. The INTER-NDA’s norms are international standards, rather than population-specific references, of early child development constructed from five low-risk, international populations according to the WHO MGRS’ prescriptive guidelines [18]. For all domains, except negative behavior, higher scores reflect better outcomes and no, any, mild-to-moderate and severe delay are defined as >10th, <10th, 3rd–10th and <3rd centiles, respectively [18]. For negative behavior, lower scores reflect better outcomes and no, any, mild-to-moderate and severe problems are defined as <90th, >90th, 90th–97th and >97th centiles, respectively [18].

### Statistical analysis

Statistical analysis was performed in IBM SPSS V.25.0 and R V4.2.0. Summary statistics for prenatal and perinatal characteristics were compared between children completing the INTER-NDA and those lost to follow-up.

The distributions of INTER-NDA and BCI outcomes were inspected visually and by using the Kolmogorov–Smirnov test. Most INTER-NDA scores and all BCIs (across all ages) were normally distributed. Associations between BCIs at each assessment age and INTER-NDA outcomes at 2 years were examined using six statistical methods:

(i) Continuous correlations.
(ii) Differences in BCIs were examined, for each INTER-NDA domain, between children with no and any delay using independent sample *t-*tests. A second analysis compared these between children with no, mild-to-moderate and severe delay using analysis of variance.
(iii) For BCIs found to be significantly associated with any delay at 2 years, receiver operating characteristic (ROC) curves were constructed and the area under the curves (AUCs) estimated.
(iv) Associations between BCIs at each assessment age and potential confounding variables were examined using continuous correlations, independent sample *t-*tests and Mann–Whitney tests as appropriate. The full list of covariates examined is presented in Table S2. This included anthropometric measures (expressed as *z* scores). These particular covariates were selected as they are the key early life indicators reported in previous scientific evidence to be associated with neurodevelopment delay during early childhood [24].
(v) Binary logistic regression analyses for factors identified in (ii) and (iii) as being associated with any delay at 2 years.
(vi) Trajectory modeling for fat% and its *z*-score values were produced using the LMS method in the *GAMLSS* package for R. Critical periods of fat accretion associated with INTER-NDA scores were examined using multiple linear regression.

We were interested to examine whether length was more strongly associated with neurodevelopmental delay than BCIs. We restricted our comparisons to the length and BCIs identified in (i), (ii) and (iv) to be associated with INTER-NDA outcomes. We quantified effect sizes using Cohen’s *d* (*d*) and 95% confidence intervals (CIs) [25].

## Results

### Characteristics of study population

**Fig. 1:**
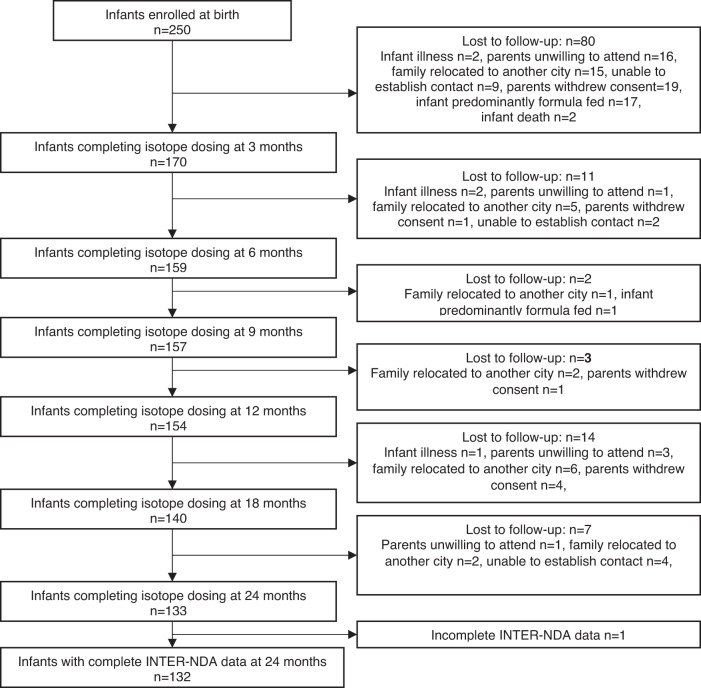
Participant flow for the Pakistan Multi-Centre Body Composition Reference Study (MBCRS) INTER-NDA: The INTERGROWTH-21st Neurodevelopmental Assessment.

Of the 250 children enrolled at birth, complete INTER-NDA data was available for 132 (52.8%) (Fig. 1). Complete INTER-NDA and BCI data was available for between 64.9 and 81.2% of children at each BCI assessment age (Table S1).

A description of the prenatal, perinatal, and postnatal characteristics of the children assessed for neurodevelopment is presented in Table 1. The mean age at INTER-NDA assessment was 25.76 months (±2.36). Fifty-two percent (*n* = 69) of the sample were boys. The cohort’s mean gestational age and weight at birth were 38.95 weeks (±1.07) and 3.13 (±0.06) kg, respectively. The overall postnatal morbidity of the cohort was low: 2.0% of children were hospitalized during the first 2 years of life with a mean hospital stay of 3.25 days (±1.89). Except for a higher rate of admissions to the neonatal unit, the baseline prenatal, perinatal and neonatal characteristics were very similar between the INTER-NDA and the attrition sample (Table 1).

**Table 1:** Prenatal, perinatal, neonatal, and postnatal characteristics in children who completed the INTER-NDA compared with those who were not assessed for neurodevelopmental outcomes at age 2 years.

	Children assessed for neurodevelopmental outcomes at 2 years (*n* = 132)	Children not assessed for neurodevelopmental outcomes at 2 years (*n* = 118)	*p* value
	Mean (SD) or number (%)	Mean (SD) or number (%)	
Mother’s age at birth (years)	28.52 (4.60)	28.44 (4.17)	0.88
Mother’s marital status at birth	Married: *n* = 131 (99.2%) Widowed: *n* = 1 (0.8%)	Married: *n* = 118 (100%) Widowed: *n* = 0	0.34
Total time the mother has spent in education (years)	14.21 (2.72)	14.59 (2.31)	0.24
Highest level of education attained by the mother	University: *n* = 77 (58.3%) Professional technical training: *n* = 20 (15.2%) Secondary education: *n* = 34 (25.7%) Primary education: *n* = 1 (0.8%)	University: *n* = 86 (72.9%) Professional technical training: *n* = 10 (8.4%) Secondary education: *n* = 22 (18.7%) Primary education: *n* = 0	0.08
Mother’s occupation	Homemaker: *n* = 108 (81.8%) Employed outside the home: *n* = 24 (18.2%)	Homemaker: *n* = 93 (81.4%) Employed outside the home: *n* = 25 (18.6%)	0.55
Mother’s weight at booking (kgs)	60.42 (11.20)	59.68 (10.70)	0.59
Number of previous births	1.03 (1.17)	0.92 (1.15)	0.44
Average monthly household income from all sources	>100,000: *n* = 45 (34.1%) 75,000–100,000: *n* = 33 (25.0%) 50,000–75,000: *n* = 54 (40.9%)	>100,000: *n* = 35 (29.7%) 75,000–100,000: *n* = 40 (33.9%) 50,000–75,000: *n* = 43 (36.4%)	0.30
Mode of delivery	Vaginal spontaneous: *n* = 74 (56.1%) Vaginal assisted (forceps or vacuum): *n* = 5 (3.8%) Cesarean section: *n* = 53 (40.1%)	Vaginal spontaneous: *n* = 70 (59.3%) Vaginal assisted (forceps or vacuum): *n* = 7 (6.0%) Cesarean section: *n* = 41 (34.7%)	0.55
Gestational age at birth (in weeks)	38.95 (1.07)	38.82 (2.67)	0.59
Newborn sex	Male: *n* = 69 (52.3%) Female: *n* = 63 (47.7%)	Male: *n* = 62 (52.5%) Female: *n* = 56 (47.5%)	0.97
Birth weight (kgs)	3.13 (0.06)	3.04 (0.46)	0.31
Birth length (cms)	49.70 (1.75)	48.48 (2.16)	0.22
Birth head circumference (cms)	34.23 (0.65)	33.58 (1.49)	0.50
APGAR score at 5 min of life	9.0 (0.0)	7.75 (2.77)	0.97
Admission to the neonatal unit required post-birth	No: *n* = 95 (71.9%) Yes: *n* = 37 (28.1%)	No: *n* = 104 (88.1%) Yes: *n* = 14 (11.9%)	0.002**
Age at neurodevelopmental assessment (months)	25.76 (2.36)	–	–
Age at weaning (months)	4.25 (2.84)	–	–
Admitted in hospital during the first 2 years of life	*n* = 4 (3.0%)	–	–
Significant morbidity during the first 2 years of life	Repeated pneumonia, acute respiratory infections or bronchiolitis: *n* = 20 (15.2%) Tuberculosis: *n* = 0 Hepatitis: *n* = 0 HIV or AIDS: *n* = 0 Malaria: *n* = 0 Cardiovascular morbidity: *n* = 0 Gastrointestinal parasitosis: *n* = 0 Repeated diarrhea: *n* = 10 (7.6%) Persistent vomiting: *n* = 1 (0.8%) Dehydration: *n* = 0	–	–

**p < 0.01.

The BCI and anthropometry characteristics for the INTER-NDA sample is presented in Table 2. Mean *z* scores for all anthropometric measurements across assessment ages were within ±1. The INTER-NDA domain scores for the sample are presented in Table S3. Six (4.5%), seven (5.3%), and eight (6.1%) children, respectively, met thresholds for cognitive, language, and gross motor delays. Of these, severe delays were observed only in cognitive (1.5%, *n* = 2) and language (3.0%, *n* = 4) domains. Thirty-seven (28.0%) and 28 (21.2%) children met thresholds for positive and negative behavior problems. Of these, severe problems were observed in 17 (12.9%) and 6 (4.5%) children. Neither INTER-NDA domain scores nor rates of delays differed between sexes (Table S3).

**Table 2:** Body composition and anthropometry characteristics of children assessed for neurodevelopmental outcomes on the INTER-NDA at age 2 years.

Age at assessment (in months)	Sample size (*n*)	FFM^a^ (kg)	FM^b^ (kg)	Fat %	Weight for length^c^	Length^c^	Weight^c^	Head circumference^c^	Mid-upper arm circumference^c^	Tricerps skin fold thickness^c^	Subscapcular skin fold thickness^c^
3	120	4.58 (0.46)	1.13 (0.34)	19.62 (4.69)	−0.26 (1.01)	−0.45 (1.01)	−0.58 (0.84)	−0.37 (0.87)	−0.31 (0.82)	0.32 (0.630	−0.09 (0.92)
6	121	5.40 (0.64)	1.70 (0.53)	23.66 (5.70)	−0.53 (0.99)	−0.37 (1.03)	−0.68 (1.01)	−0.52 (0.94)	−0.23 (0.87)	0.18 (0.72)	−0.08 (0.87)
9	108	6.40 (0.83)	1.73 (0.61)	21.13 (6.38)	−0.47 (0.90)	−0.20 (1.06)	−0.51 (0.99)	−0.50 (0.86)	−0.16 (0.88)	0.01 (0.80)	−0.01 (0.94)
12	100	7.01 (0.77)	1.77 (0.53)	20.03 (5.05)	−0.50 (0.98)	−0.35 (0.93)	−0.54 (1.00)	−0.39 (0.91)	−0.19 (0.91)	0.03 (0.77)	0.24 (0.85)
18	95	7.98 (0.95)	2.09 (0.85)	20.53 (6.56)	−0.44 (0.95)	−0.29 (1.06)	−0.46 (0.96)	−0.34 (0.95)	−0.35 (0.84)	0.14 (0.81)	0.35 (0.81)
24	108	9.04 (1.15)	2.14 (0.71)	19.06 (5.22)	−0.57 (1.03)	−0.43 (1.07)	−0.60 (1.03)	−0.53 (0.99)	−0.50 (0.09)	0.03 (0.88)	0.30 (0.89)

^a^Fat free mass.

^b^Fat mass.

^c^Presented as *Z* scores.

### BCIs and neurodevelopmental outcomes

The associations between BCIs and INTER-NDA outcomes are presented in Table S4. Overall, FFM was more strongly associated with INTER-NDA outcomes than FM. Higher FFM at 6, 9 and 24 months was associated with higher positive behavior scores at 24 months (*r* = 0.19–0.21, *p* < 0.05). Higher FFM at 3 months was associated with higher gross motor (*r* = 0.24, *p* < 0.01) and lower language scores (*r* = −0.18, *p* < 0.05). Higher fat% at 24 months was associated with lower positive behavior scores (*r* = −0.21, *p* < 0.05).

In unadjusted analyses (Table 3), compared to those without delays, children with gross motor delays had significantly lower FFM at 18 months (8.01 kg (SD 0.97) vs. 7.55 kg (SD0.20); *t* = 3.51, *p* = 0.001; Table S5). Children with positive as well as negative behavior problems had lower FFM at 6 months; children with positive behavior problems also had lower FFM at 24 months (Tables 3 and S5): these associations neared but did not achieve statistical significance (*t* = 1.73–1.98, *p* = 0.05–0.08).

**Table 3:** Unadjusted comparisons in body composition measurements between children with and without neurodevelopmental delay.

	Comparisons between any delay and no delay			Comparisons between severe, mild-to-moderate, and no delay		
	FFM	FM	Fat%	FFM	FM	Fat%
Age at body composition assessment (in months)	*p* value	*p* value	*p* value	*p* value	*p* value	*p* value
Cognition at 24 months						
3	0.10	0.81	0.06	0.62	0.26	0.21
6	0.65	0.14	0.23	0.33	0.85	0.44
9	0.59	0.89	0.58	0.79	0.63	0.75
12	0.79	0.88	0.86	0.20	0.89	0.96
18	0.37	0.38	0.30	0.27	0.58	0.36
24	0.58	0.42	0.853	0.71	0.44	0.51
Language at 24 months						
3	0.45	0.83	0.64	0.73	0.79	0.802
6	0.83	0.58	0.72	0.18	0.16	0.03*
9	0.67	0.88	0.97	0.91	0.98	0.98
12	0.62	0.65	0.51	0.88	0.90	0.81
18	0.37	0.98	0.71	0.47	0.86	0.66
24	0.30	0.66	1.00	0.51	0.90	0.98
Gross motor at 24 months						
3	0.83	0.80	0.96	0.83	0.81	0.96
6	0.63	0.20	0.31	0.63	0.20	0.37
9	0.80	0.76	0.61	0.80	0.76	0.62
12	0.47	0.59	0.55	0.47	0.59	0.55
18	0.001**	0.62	0.42	0.25	0.62	0.42
24	0.75	0.24	0.28	0.75	0.25	0.29
Positive behavior at 24 months						
3	0.38	0.42	0.38	0.63	0.17	0.14
6	0.08	0.87	0.44	0.07	0.97	0.58
9	0.62	0.30	0.46	0.21	0.40	0.37
12	0.84	0.47	0.58	0.69	0.68	0.71
18	0.45	0.50	0.86	0.19	0.79	0.70
24	0.07	0.11	0.02*	0.27	0.31	0.11
Negative behavior at 24 months						
3	0.54	0.70	0.63	0.57	0.25	0.44
6	0.05	0.43	0.93	0.17	0.75	0.98
9	0.38	0.39	0.54	0.63	0.54	0.63
12	0.58	0.91	0.74	0.58	0.92	0.93
18	0.30	0.86	0.49	0.51	0.81	0.60
24	0.09	0.11	0.03*	0.34	0.34	0.15

No children met thresholds for fine motor delays, therefore comparisons not possible for this domain. For all INTER-NDA domains, except negative behavior, no delay, any delay, severe delay and mild-to-moderate delay are defined as INTER-NDA scores >10th, <10th, <3rd and 3rd–10th centiles, respectively, on the INTER-NDA standards. For negative behavior, no problems, any problems, severe problems and mild-to-moderate problems are defined as INTER-NDA scores <90th, >90th, >97th and 90th–97th centiles, respectively, on the INTER-NDA standards. All comparisons made using the independent sample *t-*test.

*p < 0.05; **p < 0.01.

Compared to children without behavior problems, children with positive and negative behavior problems had significantly higher fat% at 24 months (20.62% (SD 4.30) vs. 18.23% (SD 5.46); *t* = −2.28, *p* = 0.02 and 20.89% (SD 4.24) vs. 18.54% (SD 5.38); *t* = −2.25, *p* = 0.03; Tables 3 and S5). Children with cognitive delay also had higher fat% at 3 months, this association neared but did not achieve statistical significance (*t* = −2.37, *p* = 0.06).

Following ROC curve analysis (Fig. S6a–c), only the association between fat% at 24 months and positive and negative behavior problems at 24 months remained significant (AUC = 0.65, 95% CI 0.54, 0.75, *p* = 0.01 and AUC = 0.64, 95% CI 0.53, 0.76; *p* = 0.03). Following binary logistic regression adjusting for covariates (Table S7), none of the BCIs identified remained significantly associated with neurodevelopmental delays across any domains (Table 4).

**Table 4:** Binary logistic regression analyses for factors associated with risk of any developmental delay at 2 years.

Dependent variable	Independent variables	*B*	S.E.	Wald	df	Sig.	Exp(*B*)	*R*^2^	Df
Cognitive delay	Age at INTER-NDA assessment	0.931	0.954	0.951	1	0.329	2.537	0.17	7
	Fat% at 3 months	0.962	0.747	1.658	1	0.198	2.618		
	Length at 18 months	2.846	3.401	0.7	1	0.403	17.218		
	Head circumference at 18 month	−9.128	8.738	1.091	1	0.296	0		
	Length at 24 months	−3.837	3.829	1.004	1	0.316	0.022		
	Head circumference at 24 month	3.046	4.623	0.434	1	0.51	21.04		
	Admission to NICU	−4.968	4.161	1.425	1	0.233	0.007		
Language delay	Age at INTER-NDA assessment	−0.889	0.473	3.535	1	0.06	0.411	0.11	5
	Admission to NICU	1.265	1.343	0.888	1	0.346	3.545		
	FFM at 3 months	−0.636	1.243	0.262	1	0.609	0.529		
	Fat% at 6 months	−0.037	0.103	0.129	1	0.72	0.964		
	Mid-upper arm circumference at 18 month	0.201	0.752	0.072	1	0.789	1.223		
Gross motor delay	Age at INTER-NDA assessment	−0.191	0.259	0.545	1	0.46	0.826	0.03	4
	FFM at 3 months	−1.569	1.65	0.905	1	0.342	0.208		
	FFM at 18 months	−0.667	0.956	0.488	1	0.485	0.513		
	Mid-upper arm circumference at 24 months	0.026	0.795	0.001	1	0.974	1.026		
Positive behavior problems	Triceps skin fold thickness at 3 months	0.944	0.465	4.113	1	0.043	2.57	0.13	7
	FFM at 6 months	−0.765	0.666	1.32	1	0.251	0.465		
	Weight at 6 months	0.26	0.459	0.32	1	0.571	1.296		
	Length at 6 months	0.231	0.43	0.288	1	0.591	1.26		
	Head circumference at 18 months	−0.365	0.404	0.819	1	0.365	0.694		
	FFM at 24 months	0.018	0.378	0.002	1	0.963	1.018		
	Fat% at 24 months	0.049	0.065	0.578	1	0.447	1.051		
Negative behavior problems	Maternal age at birth in years	−0.074	0.061	1.508	1	0.219	0.928	0.11	5
	Number of years of maternal education	−0.106	0.099	1.153	1	0.283	0.899		
	Tricpes skin fold thickness at 3 months	0.609	0.436	1.958	1	0.162	1.839		
	FFM at 6 months	−0.394	0.436	0.817	1	0.366	0.674		
	Fat% at 24 months	0.072	0.052	1.909	1	0.167	1.075		

No children met thresholds for fine motor delays, therefore regression analyses not undertaken for this domain. Covariates selected following the results presented in Tables 3 and S4 and S7.

*FFM* fat free mass, *FM* fat mass.

### Fat percentage trajectories: associations with behavior outcomes

**Fig. 2:**
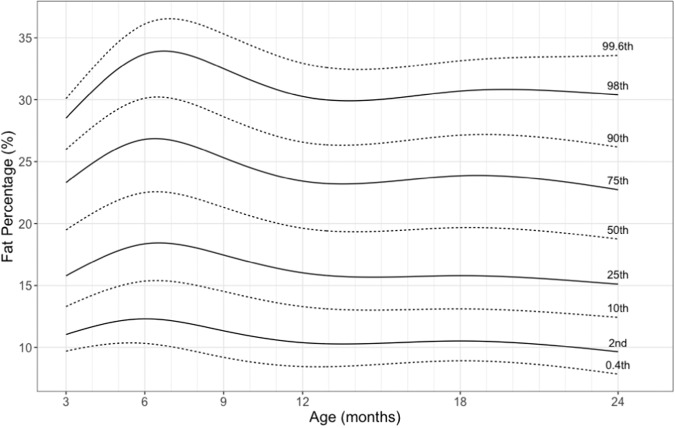
LMS centile chart for fat percentage by age.

Fat% rose from a mean value of 19.6% (SD 4.8%) at 3 months of life to 23.7% (SD 5.7%) at 6 months before falling to 19.1% (SD 5.2%) at 24 months of life (Fig. 2). The period between 12 months and 18 months was identified as a critical period of fat accretion during which an increase of 1 SD in fat% *z*-score was associated with an increase of 13.8 in negative behavior scores (Table S8). No such associations were identified for other periods or with positive behavior.

### Associations with neurodevelopment: comparisons between length, FFM and fat% accretion

The associations between length, FFM and fat% and cognitive delays, gross motor delays and behavior problems are presented in Table S9. The assessment ages and domains for these comparisons were selected following previous analyses (Tables 3 and S4 and S5). Lower length at 18 months was more strongly associated with gross motor delay than lower FFM at the same age (*d*_length_ = 0.8, 95% CI −0.01, 1.65 and *d*_FFM_ = 0.50, 95% CI −0.34, 1.31). Higher fat% at 24 months was more strongly associated with positive behavior problems than lower length at 18 months (*d*_Fat%_ = 0.52, 95% CI −0.94, −0.08 and *d*_length_ = 0.50, 95% CI 0.06, 0.94). Higher fat% at 24 months was significantly associated with negative behavior problems (*t* = −2.25, *p* −0.03) whereas length was not. Neither length, FFM nor fat% were associated with cognitive, language or fine motor delays.

## Discussion

We have shown that in healthy, term-born, breast-fed infants from Pakistan, FFM during early life is more strongly associated with neurodevelopment at 2 years than FM. These associations vary according to neurodevelopment domain and the age at which BC was assessed. Higher FFM at 6, 9 and 24 months was associated with better positive behavior scores; higher FFM at 3 months was associated with higher gross motor and lower language scores. No associations remained significant after adjusting for covariates. Behavior outcomes were particularly affected. Higher fat% at 24 months associated with poorer behavior scores at the same age, and fat accretion between 12 and 18 months representing the critical period for this association. Additionally, we have shown that length may not be a universal proxy marker for neurodevelopment during early childhood. To our knowledge, our study reports the first evidence of the association between BC and neurobehavioral outcomes in children from a LMIC. We included BC measurements between 3 and 24 months, assessed a range of neurodevelopmental outcomes at 24 months and used international standards of child development (rather than references) to categorize delay.

Our findings are consistent with reports from high-income populations that higher adiposity and/or lack of appropriate FFM growth during early life may be detrimental to developmental trajectories [8]. In a US cohort of 20 preterm and 51 full-term children, lower fat% and higher FFM gains during early life were associated with better neurocognitive outcomes at 4 years [8]. In an Italian cohort of preterm babies, FFM at term equivalent age was associated with higher language and motor composite scores at 2 years [26]. It has been suggested that FFM represents overall protein accretion, which along with growth factors such as insulin-like growth factor-1, are necessary for brain maturational and myelination processes [27].

Adiposity and FFM have been associated with preschool behavior in studies from the Netherlands [28], Sweden [15] and Switzerland [14] although the Swedish study found increased FFM to be associated with increased hyperactivity scores. Similarly, a pilot study of preterm infants from USA showed that increased FFM at 4 months was associated with higher activity levels and impulsivity, and increased FM at discharge and 4 months were associated with negative temperament at 12 and 24 months [29]. Although the mechanisms underlying these associations are not yet fully understood, the relationship has been attributed, in part, to shared risk factors, such as parenting behaviors and low socioeconomic status [28]. Unsurprisingly, in some studies of well-nourished children from high-income countries these associations are weak to lacking [11, 30].

The differential effects of assessment age on associations suggest that specific body composition changes incur neurodevelopmental benefit during differential windows of sensitivity [8]. Studies of preterm infants have shown that growth during each time period is associated with different neurocognitive changes, pointing to differential sensitive growth periods for specific brain areas resulting in differential downstream effects on the neurodevelopmental domains associated with these areas as they mature [6, 7].

Our study has some limitations. The study population may not be representative of the entire country because this was a single-center study and the cohort was recruited to be normative for BC, i.e., breast-fed and low-risk for health and nutritional morbidity, in accordance with the principal objective of the BCI. Consequently, (1) the distribution of BC was relatively homogenous and (2) the prevalence of neurodevelopmental delay was low in the cohort. It is possible that stronger associations may be observed in more heterogeneous cohorts, and those with a high-risk group (such as preterm infants). Importantly, the study was powered to detect FM and FFM and not to examine associations between BC and neurodevelopment. Therefore, despite our large overall sample size, the small number of children with delays (particularly for cognitive, language and motor outcomes) limits the generalizability of findings. This might also explain why (1) associations observed were weak and (2) certain associations approached, but did not reach, statistical significance. Moreover, as the number of covariates adjusted using multiple comparisons was large relative to the number of infants included in our analysis, it is important to consider the likelihood of type II error when interpreting our findings. We were unable to compare INTER-NDA scores between children with high and low BC indices for age due to lack of internationally valid BC thresholds for children. Finally, while our study examined associations between BC and neurodevelopment, it was not designed to examine causal relationships. Future studies in more diverse and heterogeneous populations will be important to address these issues. Our findings highlight the importance of balancing neurodevelopment and metabolic risk when designing such studies and when planning nutritional interventions for young children.

## Conclusion

We report evidence in a low-risk LMIC population that early BC indices—higher FFM and lower fat%—are associated with enhanced behavioral and gross motor outcomes at 2 years. Further research from other LMIC populations is needed to determine whether these findings are consistent across diverse populations.

## Supplementary information

Supplementary tables

## Data Availability

The datasets generated and/or analyzed during the current study are available from the corresponding author on reasonable request.
