# Supplementary material for: Differential associations between body composition indices and neurodevelopment during early life in term-born infants: findings from the Pakistan cohort: Multi-Center Body Composition Reference Study
Source: Eur J Clin Nutr. 2023 Jul 12;78(11):970–8. doi: 10.1038/s41430-023-01296-6 (PMC11537957; doi:10.1038/s41430-023-01296-6)
Supplement: Supplementary file 1 — Supplementary tables [file 41430_2023_1296_MOESM1_ESM.docx]

**SUPPORTING INFORMATION**

**S1** Study design showing time points of body composition, anthropometric and neurodevelopmental assessments.

**S2** Covariates examined for associations with neurodevelopmental outcomes at age 2 years.

**S3** Neurodevelopmental scores and rates of delay for overall sample, and for male and female children.

**S4** Correlations between growth measures (body composition and anthropometry) between 3 and 24 months and neurodevelopmental outcomes (INTER-NDA domain scores) at 24 months.

**S5** Body composition measures in children with and without neurodevelopmental delay.

**S6** Receiver operating characteristics curves for the relationship between fat free mass (18 months) and gross motor delay; and fat% at 24 months and behavioural problems.

**S7** Associations between perinatal and postnatal covariates and neurodevelopment scores at 24 months**.**

**S8** Results for multiple linear regression of associations between the change in fat z-score during different time periods and INTER-NDA negative and positive behavior scores.

**S9** Comparisons between length, FFM and Fat% and neurodevelopmental delay

|  | **Recruitment & baseline assessment** | **Age at follow Up Assessments**  **(in months)** | | | | | |
| --- | --- | --- | --- | --- | --- | --- | --- |
|  | **Birth** | **3** | **6** | **9** | **12** | **18** | **24** |
| **Body composition measurements**^a^ |  | **+** | **+** | **+** | **+** | **+** | **+** |
| **Anthropometric measurements**^b^ | **+^d^** | **+** | **+** | **+** | **+** | **+** | **+** |
| **Neurodevelopment assessment**^c^ |  |  |  |  |  |  | **+** |
| **Children with complete body composition measurements (n)** | **0** | **170** | **159** | **157** | **154** | **140** | **133** |
| **Children with complete body composition and neurodevelopment measurements (n)** | **0** | **120** | **121** | **108** | **100** | **95** | **108** |

^a^Fat free mass, fat mass and fat% measured using the Deuterium Dilution Method; ^b^Weight, length, head circumference, mid-upper arm circumference, and triceps and subscapular skin fold thickness measured according to the protocol of the WHO Multicenter Growth Reference Study; ^c^Measured on the INTERGROWTH-21^st^ Project Neurodevelopment Assessment (INTER-NDA); ^d^Weight, length and head circumference only.

**Table S1** Study design showing time points of body composition, anthropometric and neurodevelopmental assessments.

| **Socio-demographic and prenatal Covariates** | Number of years of maternal education  Average household monthly income  Number of previous births  Maternal age at birth |
| --- | --- |
| **Perinatal Covariates** | Gestational age at birth  Birth weight, length and head circumference  Mode of delivery |
| **Neonatal Covariates** | APGAR score at 5 minutes of life  Admission to neonatal unit post-birth; |
| **Postnatal Covariates** | Age at INTER-NDA assessment  Age at weaning  Hospital admissions during the first two years of life |
| **Anthropometric Covariates** | At 3, 6, 9, 12, 18, and 24 months of age:  Weight  Length  Head circumference  Mid-upper arm circumference  Triceps skin fold thickness  Subscapular skin fold thickness |

**Table S2** Covariates examined for associations with neurodevelopmental outcomes at age 2 years

|  | **Standardized INTER-NDA domain scores** | | |  | **Children with any delay** | | **Children with severe and mild-to-moderate delay** | | |
| --- | --- | --- | --- | --- | --- | --- | --- | --- | --- |
| **INTER-NDA domains** | **Total sample**  **(n=132)** | **Males**  **(n=69)** | **Females**  **(n=63)** | **P-value**^c^ | **N (%)** | **P-value**^d^ | **Mild-to-moderate delay**  **N (%)** | **Severe delay**  **N (%)** | **P-value**^e^ |
| **Cognition**^a^ | 70.29 (16.31) | 69.93 (17.28) | 70.69 (15.32) | 0.79 | 6 (4.5%) | 0.47 | 4 (3.0%) | 2 (1.5%) | 0.39 |
| **Language**^a^ | 60.32 (25.09) | 61.67 (24.98) | 58.84 (25.34) | 0.52 | 7 (5.3%) | 0.78 | 3 (2.3%) | 4 (3.0%) | 0.87 |
| **Fine Motor**^b^ | 100.00 (16.67) | 100.00 (16.67) | 100.00 (11.12) | 0.81 | 0 (0%) | - | 0 (0%) | 0 (0%) | - |
| **Gross Motor**^b^ | 88.89 (22.23) | 88.89 (22.23) | 83.34 (33.34) | 0.18 | 8 (6.1%) | 0.55 | 8 (6.1%) | 0 (0%) | - |
| **Positive Behavior**^a^ | 66.44 (23.54) | 64.06 (23.41) | 69.05 (23.60) | 0.23 | 37 (28.0%) | 0.52 | 20 (15.2%) | 17 (12.9%) | 0.74 |
| **Negative Behavior**^b^ | 25.00 (50.00) | 25.00 (38.00) | 25.00 (50.00) | 0.09 | 28 (21.2%) | 0.31 | 22 (16.7%) | 6 (4.5%) | 0.51 |

^a^For normally distributed INTER-NDA domains: Mean and SD presented, group comparisons examined using independent sample t test; ^b^For non-normally distributed INTER-NDA domains with no suitable transformation identified: Mean and IQR presented, group comparisons examined using Mann Whitney test; ^c^Comparisons in INTER-NDA scores between male and female children; ^d^Comparisons between male and female children with no delay and any delay; ^e^Comparisons between male and female children with no delay, mild-to-moderate delay and severe delay. No delays identified for fine motor domain, and no severe delay identified for gross motor domain; group comparisons therefore not performed for these domains.

**Table S3:** Neurodevelopmental scores and rates of delay for overall sample, and for male and female children.

| **INTER-NDA domains** | **FFM** | **FM** | **Fat %** | **Weight for length^c^** | **Length^c^** | **Weight^c^** | **Head Circumference^c^** | **Mid Upper Arm Circumference^c^** | **Triceps Skin Fold Thickness^c^** | **Subscapcular Skin Fold Thickness^c^** |
| --- | --- | --- | --- | --- | --- | --- | --- | --- | --- | --- |
| **Age at body composition and growth assessment: 3 months** | | | | | | | | | | |
| **Cognition**^a^ | -0.20 | 0.14 | 0.13 | -0.01 | 0.06 | 0.04 | -0.02 | 0.02 | -0.14 | -0.05 |
| **Language**^a^ | -0.18* | 0.07 | 0.12 | -0.09 | -0.08 | -0.19 | -0.14 | -0.10 | -0.16 | -0.08 |
| **Fine Motor**^b^ | 0.06 | 0.06 | 0.05 | 0.02 | 0.06 | 0.04 | 0.11 | -0.02 | -0.10 | -0.14 |
| **Gross Motor**^b^ | 0.24** | 0.09 | 0.03 | 0.07 | 0.13 | 0.17 | 0.12 | 0.08 | 0.03 | 0.11 |
| **Positive Behavior**^a^ | -0.08 | -0.02 | -0.01 | -0.13 | 0.06 | -0.06 | -0.04 | -0.12 | -0.21* | -0.09 |
| **Negative Behaviour**^b^ | 0.05 | 0.03 | 0.03 | 0.09 | -0.05 | 0.01 | 0.07 | 0.17 | 0.19* | 0.03 |
| **Age at body composition and growth assessment: 6 months** | | | | | | | | | | |
| **Cognition**^a^ | 0.09 | 0.05 | 0.01 | 0.06 | 0.07 | 0.09 | 0.11 | 0.14 | 0.16 | 0.01 |
| **Language**^a^ | -0.03 | 0.01 | 0.02 | 0.01 | -0.12 | -0.07 | -0.01 | 0.11 | 0.17 | -0.07 |
| **Fine Motor**^b^ | -0.02 | 0.09 | 0.10 | 0.06 | -0.07 | 0.01 | -0.12 | 0.07 | 0.06 | 0.03 |
| **Gross Motor**^b^ | 0.01 | 0.06 | 0.05 | 0.04 | 0.02 | 0.03 | -0.02 | 0.09 | -0.02 | -0.11 |
| **Positive Behavior**^a^ | 0.19* | 0.04 | -0.03 | 0.03 | 0.18 | 0.13 | 0.05 | 0.08 | 0.09 | 0.03 |
| **Negative Behaviour**^b^ | -0.05 | -0.07 | -0.03 | -0.06 | -0.07 | -0.08 | -0.11 | -0.05 | -0.13 | -0.07 |
| **Age at body composition and growth assessment: 9 months** | | | | | | | | | | |
| **Cognition**^a^ | 0.03 | 0.04 | 0.04 | -0.06 | 0.18 | 0.06 | 0.15 | 0.10 | 0.12 | -0.03 |
| **Language**^a^ | -0.06 | 0.01 | 0.03 | -0.09 | -0.02 | -0.08 | -0.01 | -0.01 | 0.13 | -0.04 |
| **Fine Motor**^b^ | -0.08 | 0.13 | 0.16 | 0.02 | 0.03 | 0.04 | -0.06 | 0.06 | 0.03 | 0.03 |
| **Gross Motor**^b^ | 0.05 | 0.02 | 0.01 | 0.03 | 0.02 | 0.03 | 0.01 | 0.11 | -0.03 | -0.08 |
| **Positive Behavior**^a^ | 0.23* | 0.06 | -0.03 | 0.07 | 0.28** | 0.20* | 0.11 | 0.18 | 0.17 | 0.04 |
| **Negative Behaviour**^b^ | -0.11 | -0.09 | -0.06 | -0.07 | -0.13 | -0.13 | -0.10 | -0.09 | -0.08 | -0.02 |
| **Age at body composition and growth assessment: 12 months** | | | | | | | | | | |
| **Cognition**^a^ | 0.06 | 0.06 | 0.05 | -0.01 | 0.15 | 0.06 | 0.12 | 0.15 | -0.02 | -0.09 |
| **Language**^a^ | -0.05 | -0.01 | 0.02 | -0.10 | -0.08 | -0.01 | 0.14 | 0.11 | --0.18 | -0.13 |
| **Fine Motor**^b^ | 0.04 | 0.12 | 0.12 | 0.14 | 0.02 | 0.15 | 0.06 | 0.23* | -0.08 | -0.11 |
| **Gross Motor**^b^ | 0.14 | -0.02 | -0.05 | 0.07 | 0.01 | 0.07 | 0.01 | 0.06 | -0.07 | 0.02 |
| **Positive Behavior**^a^ | 0.03 | 0.05 | 0.03 | 0.03 | 0.07 | 0.04 | 0.06 | 0.04 | 0.04 | 0.03 |
| **Negative Behaviour**^b^ | -0.17 | -0.02 | 0.02 | -0.09 | -0.09 | -0.09 | -0.11 | -0.02 | -0.07 | -0.06 |
| **Age at body composition and growth assessment: 18 months** | | | | | | | | | | |
| **Cognition**^a^ | 0.04 | 0.17 | 0.12 | -0.01 | 0.23* | 0.12 | 0.24* | 0.10 | 0.03 | 0.04 |
| **Language**^a^ | -0.02 | 0.03 | -0.01 | -0.03 | -0.08 | -0.06 | 0.06 | 0.20* | 0.06 | -0.07 |
| **Fine Motor**^b^ | 0.08 | -0.08 | -0.08 | 0.06 | 0.07 | -0.04 | 0.20* | 0.01 | -0.06 | -0.06 |
| **Gross Motor**^b^ | 0.06 | -0.06 | -0.07 | 0.03 | 0.07 | 0.03 | 0.01 | -0.13 | -0.13 | -0.16 |
| **Positive Behavior**^a^ | 0.05 | 0.11 | 0.05 | -0.04 | 0.20 | 0.07 | 0.21* | -0.11 | -0.03 | 0.02 |
| **Negative Behaviour**^b^ | -0.15 | 0.03 | 0.07 | -0.09 | -0.20 | -0.14 | -0.11 | -0.06 | -0.06 | -0.62 |
| **Age at body composition and growth assessment: 24 months** | | | | | | | | | | |
| **Cognition**^a^ | 0.12 | 0.05 | -0.01 | -0.08 | 0.22* | 0.07 | 0.22* | -0.05 | -0.08 | -0.09 |
| **Language**^a^ | 0.03 | -0.07 | -0.08 | -0.15 | 0.05 | -0.08 | 0.06 | -0.09 | -0.09 | -0.12 |
| **Fine Motor**^b^ | 0.11 | 0.05 | 0.02 | 0.03 | 0.14 | 0.11 | 0.07 | 0.19 | 0.02 | 0.02 |
| **Gross Motor**^b^ | 0.15 | 0.03 | -0.01 | 0.14 | 0.09 | 0.13 | 0.08 | 0.27** | 0.02 | 0.02 |
| **Positive Behavior**^a^ | 0.21* | -0.15 | -0.21* | 0.04 | 0.06 | 0.06 | 0.10 | -0.09 | -0.03 | 0.08 |
| **Negative Behaviour**^b^ | -0.18 | 0.09 | 0.14 | -0.04 | -0.06 | -0.10 | -0.14 | -0.05 | -0.18 | -0.18 |

**p<0.01; *p<0.05.

FFM: Fat free mass; FM: Fat mass

^a^For normally distributed INTER-NDA domains: Associations examined using Pearsons correlations; ^b^For non-normally distributed INTER-NDA domains: Associations examined using Spearmans correlations, ^c^Presented as z scores.

**Table S4** Correlations between growth measures (body composition and anthropometry) between 3 and 24 months and neurodevelopmental outcomes (INTER-NDA domain scores) at 24 months.

|  | | **COGNITION** | | | **LANGUAGE** | | | **GROSS MOTOR** | | | **POSITIVE BEHAVIOUR** | | | **NEGATIVE BEHAVIOUR** | | |
| --- | --- | --- | --- | --- | --- | --- | --- | --- | --- | --- | --- | --- | --- | --- | --- | --- |
| **Age at body composition assessment** | **Presence of delay** | **N** | **Mean** | **SD** | **N** | **Mean** | **SD** | **N** | **Mean** | **SD** | **N** | **Mean** | **SD** | **N** | **Mean** | **SD** |
| **3 months** | | | | | | | | | | | | | | | | |
| FFM | No Delay | 115 | 4.59 | 0.47 | 112.00 | 4.60 | 0.47 | 115.00 | 4.59 | 0.46 | 87.00 | 4.56 | 0.44 | 93.00 | 4.57 | 0.45 |
|  | Any Delay | 5 | 4.54 | 0.36 | 7.00 | 4.46 | 0.17 | 5.00 | 4.54 | 0.51 | 33.00 | 4.65 | 0.53 | 27.00 | 4.64 | 0.50 |
| FM | No Delay | 115 | 1.12 | 0.33 | 112.00 | 1.13 | 0.34 | 115.00 | 1.13 | 0.34 | 87.00 | 1.14 | 0.35 | 93.00 | 1.14 | 0.34 |
|  | Any Delay | 5 | 1.37 | 0.29 | 7.00 | 1.16 | 0.32 | 5.00 | 1.09 | 0.35 | 33.00 | 1.09 | 0.30 | 27.00 | 1.11 | 0.33 |
| Fat% | No Delay | 115 | 19.47 | 4.80 | 112.00 | 19.54 | 4.83 | 115.00 | 19.62 | 4.75 | 87.00 | 19.85 | 4.82 | 93.00 | 19.74 | 4.76 |
|  | Any Delay | 5 | 23.05 | 3.22 | 7.00 | 20.42 | 4.64 | 5.00 | 19.52 | 6.20 | 33.00 | 19.00 | 4.71 | 27.00 | 19.21 | 4.94 |
| **6 months** | | | | | | | | | | | | | | | | |
| FFM | No Delay | 117 | 5.42 | 0.63 | 114.00 | 5.41 | 0.64 | 114.00 | 5.40 | 0.64 | 88.00 | 5.47 | 0.63 | 94.00 | 5.46 | 0.65 |
|  | Any Delay | 4 | 4.95 | 0.69 | 6.00 | 5.36 | 0.62 | 7.00 | 5.52 | 0.70 | 33.00 | 5.24 | 0.64 | 27.00 | 5.21 | 0.56 |
| FM | No Delay | 117 | 1.69 | 0.53 | 114.00 | 1.71 | 0.53 | 114.00 | 1.68 | 0.51 | 88.00 | 1.69 | 0.53 | 94.00 | 1.72 | 0.53 |
|  | Any Delay | 4 | 1.81 | 0.41 | 6.00 | 1.58 | 0.56 | 7.00 | 1.94 | 0.78 | 33.00 | 1.71 | 0.52 | 27.00 | 1.63 | 0.50 |
| Fat% | No Delay | 117 | 23.54 | 5.65 | 114.00 | 23.72 | 5.60 | 114.00 | 23.53 | 5.53 | 88.00 | 23.40 | 5.57 | 94.00 | 23.69 | 5.59 |
|  | Any Delay | 4 | 27.03 | 7.09 | 6.00 | 22.87 | 8.36 | 7.00 | 25.76 | 8.27 | 33.00 | 24.34 | 6.05 | 27.00 | 23.56 | 6.16 |
| **9 months** | | | | | | | | | | | | | | | | |
| FFM | No Delay | 103 | 6.40 | 0.84 | 101.00 | 6.42 | 0.85 | 100.00 | 6.39 | 0.85 | 76.00 | 6.43 | 0.78 | 83.00 | 6.44 | 0.84 |
|  | Any Delay | 5 | 6.45 | 0.74 | 6.00 | 6.27 | 0.24 | 8.00 | 6.47 | 0.63 | 32.00 | 6.33 | 0.96 | 25.00 | 6.27 | 0.80 |
| FM | No Delay | 103 | 1.74 | 0.62 | 101.00 | 1.73 | 0.62 | 100.00 | 1.73 | 0.59 | 76.00 | 1.77 | 0.58 | 83.00 | 1.76 | 0.58 |
|  | Any Delay | 5 | 1.59 | 0.39 | 6.00 | 1.69 | 0.56 | 8.00 | 1.67 | 0.85 | 32.00 | 1.63 | 0.67 | 25.00 | 1.63 | 0.71 |
| Fat% | No Delay | 103 | 21.21 | 6.50 | 101.00 | 21.10 | 6.45 | 100.00 | 21.22 | 6.26 | 76.00 | 21.46 | 5.90 | 83.00 | 21.37 | 5.93 |
|  | Any Delay | 5 | 19.60 | 2.67 | 6.00 | 21.02 | 5.83 | 8.00 | 20.05 | 8.11 | 32.00 | 20.36 | 7.43 | 25.00 | 20.34 | 7.75 |
| **12 months** | | | | | | | | | | | | | | | | |
| FFM | No Delay | 96 | 7.01 | 0.76 | 94.00 | 7.03 | 0.79 | 93.00 | 7.02 | 0.79 | 72.00 | 7.02 | 0.73 | 78.00 | 7.03 | 0.76 |
|  | Any Delay | 4 | 7.06 | 1.17 | 5.00 | 6.85 | 0.32 | 7.00 | 6.81 | 0.43 | 28.00 | 6.98 | 0.89 | 22.00 | 6.92 | 0.83 |
| FM | No Delay | 96 | 1.78 | 0.54 | 94.00 | 1.77 | 0.54 | 93.00 | 1.77 | 0.51 | 72.00 | 1.80 | 0.55 | 78.00 | 1.77 | 0.53 |
|  | Any Delay | 4 | 1.71 | 0.14 | 5.00 | 1.88 | 0.38 | 7.00 | 1.88 | 0.81 | 28.00 | 1.72 | 0.49 | 22.00 | 1.78 | 0.54 |
| Fat% | No Delay | 96 | 20.04 | 5.15 | 94.00 | 19.98 | 5.13 | 93.00 | 19.94 | 4.87 | 72.00 | 20.19 | 5.19 | 78.00 | 19.92 | 4.83 |
|  | Any Delay | 4 | 19.60 | 1.38 | 5.00 | 21.49 | 3.72 | 7.00 | 21.13 | 7.44 | 28.00 | 19.59 | 4.72 | 22.00 | 20.38 | 5.88 |
| **18 months** | | | | | | | | | | | | | | | | |
| FFM | No Delay | 91 | 7.96 | 0.94 | 89.00 | 8.01 | 0.96 | 89.00 | 8.01 | 0.97 | 65.00 | 8.03 | 1.00 | 73.00 | 8.04 | 0.95 |
|  | Any Delay | 4 | 8.38 | 1.14 | 5.00 | 7.62 | 0.55 | 6.00 | 7.55 | 0.20 | 30.00 | 7.88 | 0.83 | 22.00 | 7.80 | 0.95 |
| FM | No Delay | 91 | 2.11 | 0.87 | 89.00 | 2.10 | 0.88 | 89.00 | 2.08 | 0.86 | 65.00 | 2.13 | 0.97 | 73.00 | 2.09 | 0.93 |
|  | Any Delay | 4 | 1.72 | 0.44 | 5.00 | 2.09 | 0.37 | 6.00 | 2.26 | 0.82 | 30.00 | 2.02 | 0.52 | 22.00 | 2.12 | 0.59 |
| Fat% | No Delay | 91 | 20.68 | 6.61 | 89.00 | 20.50 | 6.72 | 89.00 | 20.39 | 6.59 | 65.00 | 20.60 | 7.30 | 73.00 | 20.30 | 6.91 |
|  | Any Delay | 4 | 17.19 | 4.96 | 5.00 | 21.60 | 4.04 | 6.00 | 22.62 | 6.33 | 30.00 | 20.38 | 4.69 | 22.00 | 21.28 | 5.32 |
| **24 months** | | | | | | | | | | | | | | | | |
| FFM | No Delay | 103 | 9.06 | 1.16 | 101.00 | 9.08 | 1.16 | 100.00 | 9.05 | 1.17 | 75.00 | 9.16 | 1.22 | 84.00 | 9.12 | 1.20 |
|  | Any Delay | 5 | 8.63 | 0.98 | 6.00 | 8.58 | 0.73 | 8.00 | 8.91 | 0.97 | 33.00 | 8.77 | 0.94 | 24.00 | 8.74 | 0.90 |
| FM | No Delay | 103 | 2.15 | 0.71 | 101.00 | 2.16 | 0.72 | 100.00 | 2.17 | 0.71 | 75.00 | 2.08 | 0.73 | 84.00 | 2.09 | 0.73 |
|  | Any Delay | 5 | 1.98 | 0.56 | 6.00 | 2.02 | 0.57 | 8.00 | 1.87 | 0.67 | 33.00 | 2.30 | 0.62 | 24.00 | 2.33 | 0.60 |
| Fat% | No Delay | 103 | 19.08 | 5.25 | 101.00 | 19.07 | 5.28 | 100.00 | 19.21 | 5.20 | 75.00 | 18.38 | 5.46 | 84.00 | 18.54 | 5.38 |
|  | Any Delay | 5 | 18.63 | 5.16 | 6.00 | 19.07 | 5.13 | 8.00 | 17.16 | 5.40 | 33.00 | 20.62 | 4.30 | 24.00 | 20.89 | 4.24 |

FFM: Fat free mass; FM: Fat mass

No children met thresholds for fine motor delays, therefore comparisons not undertaken for this domain. For all INTER-NDA domains, except negative behavior, no and any delay are defined as INTER-NDA scores >10^th^ and <10^th^centiles, respectively, on the INTER-NDA standards. For negative behavior, no and any problems are defined as INTER-NDA scores <90th^th^ and >90^th^ centiles, respectively, on the INTER-NDA standards.

**S5** Body composition measures in children with and without neurodevelopmental delay.

**Figure S6** Receiver operating characteristics (ROC) curves for the relationships between (i) fat free mass (18 months) and gross motor delay and (ii) fat% at 24 months and behavior problems.

| 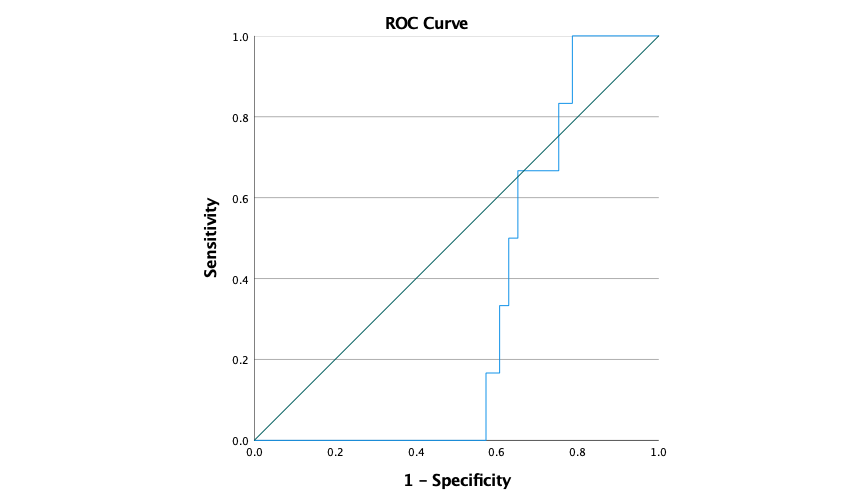 | 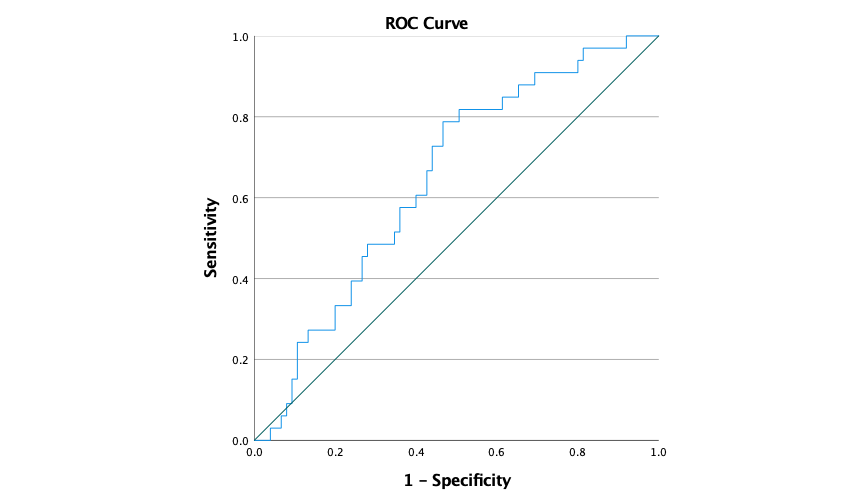 | 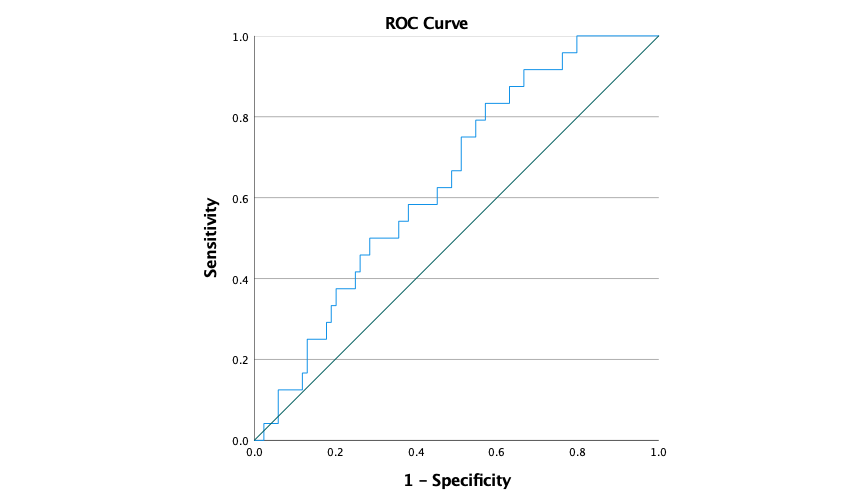 |
| --- | --- | --- |
| **Figure S6(a)** Receiver-operating characteristic (ROC) curve for relationship between FFM at 18 months and *any* gross motor delay —sensitivity vs. 1- specificity. Area under the curve=0.33 (95% CI 0.22, 0.44; p=0.017). Reference line indicates the performance of a test with no discrimination ability between delay and no delay above that of chance. | **Figure S6(b)** Receiver-operating characteristic (ROC) curve for relationship between Fat% at 24 months and *any* positive behavior problem —sensitivity vs. 1- specificity. Area under the curve=0.65 (95% CI 0.54, 0.75; p=0.01). Reference line indicates the performance of a test with no discrimination ability between delay and no delay above that of chance. | **Figure S6(c)** Receiver-operating characteristic (ROC) curve for relationship between Fat% at 24 months and *any* negative behavior problem —sensitivity vs. 1- specificity. Area under the curve=0.64 (95% CI 0.53, 0.76; p=0.03). Reference line indicates the performance of a test with no discrimination ability between delay and no delay above that of **chance.** |

| **INTER-NDA domains** | **Age at Follow-Up** | **Gestation-al Age at Birth** | **Birth Weight** | **Birth Length** | **Birth Head Circumference** | **APGAR at 5 minutes** | **Admission to Neonatal Unit Post-Birth** | **Maternal Age at Birth** | **Number of Years of Maternal Education** | **Average Household Monthly Income** | **Mode of Delivery** | **Number of Previous Births** | **Age at Weaning** | **Number of Days Spent in Hospital During First 2 Years of Life** |
| --- | --- | --- | --- | --- | --- | --- | --- | --- | --- | --- | --- | --- | --- | --- |
| **Cognition**^a^ | r=0.31** | r=-0.09 | r=-0.04 | r=0.01 | r=-0.13 | r=-0.15 | t=-3.32** | r=-0.01 | r=0.08 | F=1.84 | F=2.05 | r=-0.01 | 0.01 | 0.27 |
| **Language**^a^ | r=0.39** | r=-0.10 | r=-0.02 | r=0.01 | r=-0.17 | r=-0.11 | T=-3.822,** | r=0.08 | r=0.15 | F=1.10 | F=2.13 | r=-0.15 | 0.11 | -0.11 |
| **Fine Motor**^b^ | r=0.19* | r=-0.04 | r=0.20* | r=0.09 | r=0.07 | r=0.09 | U=2191.00* | r=0.02 | r=-0.03 | F=3.24* | F=2.00 | r=-.05 | 0.14 | 0.11 |
| **Gross Motor**^b^ | r=0.22* | r=-0.05 | r=0.07 | r=0.13 | r=-0.01 | r=0.09 | U=2045.50 | r=-0.04 | r=-0.05 | F=1.00 | F=0.82 | r=-0.01 | 0.09 | 0.50 |
| **Positive Behavior**^a^ | r=0.11 | r=-0.02 | r=0.02 | r=0.01 | r=-0.09 | r=-0.03 | T=-0.64 | r=0.08 | r=0.13 | F=2.02 | F=1.67 | r=-0.12 | -0.08 | -0.62 |
| **Negative Behaviour**^b^ | r=-0.10 | r=0.02 | r=-0.09 | r=-0.03 | r=-0.02 | r=-0.06 | U=1844.00 | r=-0.18* | r=-0.18* | F=1.99 | F=1.75 | r=0.01 | -0.08 | -0.06 |

***p<0.001; **p<0.01; *p<0.05.

^a^For normally distributed INTER-NDA domains: Associations between continuous variables examined using Pearsons correlations, group comparisons examined using independent sample t test and ANOVAs; ^b^For non-normally distributed INTER-NDA domains: Associations between continuous variables examined using Spearmans correlations, group comparisons examined using Mann Whitney test and ANOVAs.

**Table S7** Associations between perinatal and postnatal covariates and neurodevelopment scores at 24 months**.**

| **Period** | **Effect of Change in Fat Z-score on Negative Behaviour Score** | **p-value** | **Effect of Change in Fat Z-score on Positive Behaviour Score** | **p-value** |
| --- | --- | --- | --- | --- |
| 3 to 6 months | -2.2 | 0.6 | -3.0 | 0.3 |
| 6 to 9 months | 4.7 | 0.3 | -0.6 | 0.9 |
| 9 to 12 months | 9.8 | 0.1 | -6.9 | 0.1 |
| 12 to 18 months | 13.8 | 0.006** | -6.2 | 0.07 |
| 18 to 24 months | 4.4 | 0.3 | -3.4 | 0.3 |

**p<0.01

**Table S8** Results for multiple linear regression of associations between the change in fat z-score during different time periods and INTER-NDA negative and positive behavior scores.

|  | **Cognitive Delay** | | **Gross Motor Delay** | | **Positive Behaviour Problems** | | **Negative Behaviour Problems** | |
| --- | --- | --- | --- | --- | --- | --- | --- | --- |
| Growth measures | t | Sig. | t | Sig. | t | Sig. | t | Sig. |
| Length at 9 months | 1.64 | 0.17 | 1.80 | 0.11 | 1.53 | 0.13 | 1.13 | 0.26 |
| Length at 18 months | 0.86 | 0.45 | 3.12 | 0.02* | 2.36 | 0.02* | 1.12 | 0.27 |
| Length at 24 months | 1.95 | 0.11 | 2.50 | 0.04 | 0.22 | 0.82 | -0.30 | 0.76 |
| FFM at 18 months | -0.73 | 0.52 | 3.51 | <0.001** | 0.76 | 0.45 | 1.04 | 0.30 |
| Fat% at 24 months | 0.19 | 0.86 | 1.04 | 0.33 | -2.29 | 0.03* | -2.25 | 0.03* |

**p<0.01; *p<0.05.

FFM: Fat free mass. Independent t test used for comparisons.

**Table S9** Comparisons between length, FFM and Fat% and neurodevelopmental delay
